# Direct oral anticoagulants and the risk of adverse clinical outcomes among patients with different body weight categories: a large hospital-based study

**DOI:** 10.1007/s00228-023-03593-2

**Published:** 2023-11-18

**Authors:** Ezekwesiri Michael Nwanosike, Hamid A. Merchant, Wendy Sunter, Muhammad Ayub Ansari, Barbara R. Conway, Syed Shahzad Hasan

**Affiliations:** 1 Department of Pharmacy, School of Applied Sciences, University of Huddersfield, Queensgate, Huddersfield, HD1 3DH UK; 2 Department for Bioscience, School of Health, Sport and Bioscience, the University of East London, London, E16 2RD UK; 3 Calderdale and Huddersfield Pharmacy Services, Anticoagulation Services, Calderdale and Huddersfield NHS Foundation Trust Hospitals, Lindley, Huddersfield, HD3 3EA UK; 4 School of Computing and Engineering, University of Huddersfield, Queensgate, Huddersfield, HD1 3DH UK

**Keywords:** Electronic health records, Direct oral anticoagulants, Obesity, Mortality, England, Machine learning

## Abstract

**Objective:**

Through predictable pharmacokinetics—including a convenient fixed-dose regimen, direct oral anticoagulants (DOACs) are preferred over previous treatments in anticoagulation for various indications. However, the association between higher body weight and the risk of adverse consequences is not well studied among DOAC users. We aim to explore the association of body weight and adverse clinical outcomes in DOAC users.

**Methods:**

A total of 97,413 anonymised DOAC users in a tertiary care setting were identified following structured queries on the electronic health records (EHRs) to extract the feature-rich anonymised dataset. The prepared dataset was analysed, and the features identified with machine learning (ML) informed the adjustments of covariates in the multivariate regression analysis to examine the association. Kaplan–Meier analysis was performed to evaluate the mortality benefits of DOACs.

**Results:**

Among DOAC users, the odds of adverse clinical outcomes, such as clinically relevant non-major bleeding (CRNMB), ischaemic stroke, all-cause mortality, and prolonged hospital stay, were lower in patients with overweight, obesity, or morbid obesity than in patients with normal body weight. The odds of ischaemic stroke (OR 0.42, 95% CI: 0.36–0.88, *p* = 0.001) and all-cause mortality (OR 0.87, 95% CI: 0.81–0.95, *p* = 0.001) were lower in patients with morbid obesity than in patients with normal body weight. In the Kaplan–Meier analysis, apixaban was associated with a significantly lower rate of mortality overall and in obesity and overweight subgroups than other DOACs (*p* < 0.001). However, rivaroxaban performed better than apixaban in the morbid obesity subgroup (*p* < 0.001).

**Conclusion:**

This study shows the positive effects of DOAC therapy on clinical outcomes, particularly in patients with high body weight. However, this still needs validation by further studies particularly among patients with morbid obesity.

**Supplementary Information:**

The online version contains supplementary material available at 10.1007/s00228-023-03593-2.

## Introduction

Direct oral anticoagulants (DOACs) have become the mainstay for stroke prophylaxis in atrial fibrillation (AF) and the management of venous thromboembolism (VTE). Apixaban, rivaroxaban, edoxaban, and dabigatran are preferred for their advantages over conventional anticoagulants (such as warfarin) which include predictable pharmacokinetics—including a convenient fixed-dose regimen—and minimal monitoring requirements. Other notable advantages of DOACs are the wider therapeutic window and fewer interactions—including non-inferior safety and efficacy profile when compared to warfarin [1, 2].

The limitations of DOACs for special populations such as underweight patients and morbidly obese patients are also well known. The fixed doses make it difficult to adjust the dosing for optimal outcomes; besides, there is a scarcity of clinical data on safety and efficacy due to insufficient sample size in clinical studies (notably, the landmark DOAC trials) [2–4]. Given the uncertainty among prescribers due to these limitations, several guidelines have been put in place. For example, the International Society of Thrombosis and Haemostasis Scientific Standardisation Committee (ISTH SSC) restricted the prescribing of DOACs for patients who have a body mass index (BMI) greater than 40 kg/m^2^ (warfarin is recommended instead) unless results of clinical monitoring suggest otherwise [5].

The rising volume of DOAC prescriptions for extreme weight patients reflects their soaring cases globally [6]. For these cohorts, optimising outcomes is a complicated balancing act as clinicians prefer to prescribe DOACs with caution to minimise the risk of negative outcomes like strokes or bleeding events [7]. Therefore, there is a need for reassuring evidence of the clinical benefit of the DOAC types. This evidence can be derived from robust clinical studies based on sufficiently large datasets. Full consensus on DOAC prescribing for patients from different BMI classes has not been reached yet due to conflicting evidence [7–11]. Some authors suggest clinicians are reverting to alternatives like warfarin when doubt about risk/benefit arises [12, 13].

Stakeholders realise the importance of observational (real-world) studies regarding cost-effectiveness and representation of the relevant population [14]. A strong enabler of observational studies is the availability of electronic health records (EHR), for analysis—they are vast repositories of analysable data (e.g., clinical, medication, and demographic data) from patients. With sound techniques and powerful statistical models, key insights can be mined from data (provided it is of good quality).

Optimising the dose regimen of DOAC therapy requires detailed knowledge of the real-world outcomes in a sufficient sample of patients encompassing a wide range of demographics and clinical backgrounds on different DOACs. This study aims to explore the association between patients’ body weight and adverse clinical outcomes in patients on DOAC therapy by analysing EHRs using data-driven approaches with excellent pattern recognition and predictive abilities.

## Material and methods

### Study design and population

A large, retrospective, hospital-based cohort study sampled adult patients receiving DOACs in Calderdale and Huddersfield NHS Foundation Trust Hospitals in West Yorkshire, England. From the electronic health records (EHR)—accessed from Huddersfield Royal Infirmary—we identified anonymised patients admitted directly (or transferred) to the hospitals between 1^st^ May 2017 and 31^st^ October 2021 who were on DOAC therapy for the (1) management or prevention of ischaemic stroke in AF, (2) treatment and prevention of VTE (deep vein thrombosis or pulmonary embolism). Patients represented in the electronic health record (EHR) were drawn from different wards across the hospitals. Notably, we excluded outpatients, surgical/elective patients, and patients admitted to the maternity ward receiving short-term DOAC therapy. All patients who met the inclusion criteria were sampled from general medicine, the elderly, cardiology, respiratory medicine, and stroke wards.

### The electronic health records – data extraction

The EHR refers to the comprehensive (all-in-one-place) digital record of patient history, demographics, clinical profile (including biomarkers), laboratory profile, medication information, multidisciplinary treatment plan, and relevant documentation accessible to authorised personnel. The IT officer (interface and intelligence lead for information management) from *The Health Informatics Service* (THIS)—engaged by the Anticoagulation Pharmacist—facilitated data access and then applied structured queries on the EHR to extract the feature-rich dataset (reports) meeting eligibility criteria for further data analysis. Extracted data were anonymised and pre-processed (cleaned) to ensure it was in a suitable format for analysis. Categorical features like gender, race, bleeding/bleeding risk, and stroke/stroke risk were encoded using the label encoding method. Feature engineering (i.e., the systematic selection of predictive features) was informed by the domain expertise of clinical pharmacists. The number of features was trimmed (redundant or unnecessary features were excluded), and some features were modified, making them more informative.

Given that most patients had several events (treatment episodes), we chose the last treatment (dose of medication) the patient received (last treatment encounter). This reflects the stable or maintenance dose. Also, we only considered patients who received uniform treatment throughout; patients whose treatment was switched were excluded.

### Study outcomes and covariates

The outcomes included all-cause mortality (deceased), length of stay (in days), clinically relevant non-major bleeding (CRNMB) event(s) (in AF and non-surgical patients), ischaemic stroke, any thromboembolic events, and the number of emergency admissions (any hospitalisations post-DOAC treatment). We used the ISTH definition of CRNMB, which is ‘any sign or symptom of haemorrhage requiring medical intervention by a healthcare professional or leading to hospitalisation or increased level of care or prompting a face-to-face evaluation [15]. The primary outcomes were CRNMB, all-cause mortality, ischaemic stroke, and any thromboembolic events, while secondary outcomes were the length of stay and the number of emergency visits.

We set out to compare outcomes across the BMI categories. BMI (calculated by dividing weight in kg by the square of height in meters) was the body size descriptor adopted in the study; BMI classification followed the National Institute for Health and Care Excellence (NICE)/ National Health Service (NHS) standard. This was encoded accordingly, i.e., less than 18.5 (underweight) = 1; 18.5–24.9 (normal) = 2; 25–29.9 (overweight) = 3; 30–39.9 (obese) = 4; 40 and above (severe/morbid obesity) = 5.

Demographic, medication, and clinical profiles were extracted from the computerised EHR as continuous or categorical features (potential predictor variables) for each patient. Demographic data included date of treatment, age, gender, race (ethnicity), height, and weight. The medication profile comprised medication administered (apixaban, rivaroxaban, edoxaban, and dabigatran), medication dose, and a therapeutic indication of medication (according to the local formulary). The clinical profile included bleeding risk and VTE risk (calculated from local CHFT algorithm), estimated glomerular filtration rate (eGFR calculated with CKD-EPI formula)/chronic kidney disease (CKD) status, and comorbidities.

## Statistical analyses

The Statistical Package for Social Sciences (SPSS^®^ version 27) was used for data analysis. Preliminary statistical techniques were applied to test the normality in the distribution of the variables extracted for the study. Descriptive statistics were used to summarise the continuous and categorical variables (predictors and outcomes) and carry out the intra-cohort comparisons (cross-tabulation). Pearson’s test was used to measure the correlation between BMI and continuous outcomes. The Chi-Squared test was used to compare the categorical groups, for example, BMI categories versus stroke events (yes or no).

### Machine learning modelling

The ML experiments were performed on a high-performance computing (HPC) machine with 64 gigabytes (GB) RAM and a Core i5 CPU at 4.10 GHz. For machine learning, the dataset was pre-processed and normalised. Following 70%:30% partitioning of the overall dataset via stratified sampling, the selected ML classifiers (e.g., random forest, decision trees) were applied to the training dataset (70%). The categorical attributes were converted into numerical attributes using labels and one-hot encodings. Six classification and regression ML models were employed to analyse the data; this included: decision trees, K nearest neighbours, random forests, logistic regression, gradient boosting classifier, and support vector machine models.

ML applications in safety-critical sectors such as clinical medicine have less tolerance for wrong predictions and could result in catastrophic incidents. Therefore, we also explored our models’ internal workings and analysed their decision-making process. To understand the underlying decision-making process of ML models, input features and their contribution towards clinical outcomes was also analysed.

Six classification and regression ML models (decision trees, random forest, KNNs, logistic regression, gradient boosting classifier, and SVM) were trained to predict different clinical outcomes. Of these, decision trees and random forest accurately predicted the outcome (98.2% and 99.2%, respectively) with good precision metrics, such as recall precision and F1 scores, as summarised in Tables S1 and S2. Due to their superior performance, these two models were then used. Both models achieved excellent results. Support metrics represent the total number of records in the dataset corresponding to each outcome. Some of the outcomes have an equal number of records. For instance, the mortality event had 14,867 patient records that did not die, and an almost equal number of patients (14,357) died. However, the remaining events have an unequal class distribution. Judging an ML model solely on accuracy metrics could be misleading, particularly when the data is imbalanced. Despite the imbalanced data, the models exhibited excellent precision, recall, and f1-score, mainly due to the high quality of the training dataset. Both models learned the patterns correctly from the data and produced unbiased and generalised results.

### Multivariate regression

Significant variables (important features) identified from machine learning were then used to adjust the multivariate regression model. Multivariate regression with a backward stepwise approach was used, and the model was selected based on the Hosmer & Lemeshow test. Using multivariate regression, the relationship between BMI and the continuous outcomes was explored multilaterally, adjusting for covariates such as age, gender, ethnic group, comorbidity, bleeding, stroke, thrombosis, treatment years, and length of stay or emergency visit. Similarly, multivariate logistic regression was applied for categorical outcomes (e.g., mortality or stroke event), and results were presented as Odds Ratios (ORs). The odds ratio above one implied that exposure to the specific BMI class increased the odds of the outcome, while OR below one implied that exposure to the specific BMI class decreased the odds of the outcome. Kaplan–Meier analysis was performed to evaluate the significance of DOACs therapy on mortality outcome.

## Results

A total of 97,413 patients on DOAC therapy were extracted from the EHR. Twenty-four different features were selected, and additionally, six new features were created following the conversion of some continuous features to categorical features to produce a detailed statistical analysis. Table 1 shows the summary statistics of the sample variables. The mean age of the study sample was 78.8 ± 11.5 years, with an almost similar number of male and female patients. The majority of the patients were of white origin (93%) and had comorbid conditions (84%). Only 4.4% of the patients (*n* = 4,275) were morbidly obese.

**Table 1:** Characteristics of the study sample (*n* = 97,413)

**Variable**	**Total patients** **n (%)**
**Age** (yr), Mean ± SD	78.8 ± 11.5
**Gender** (% Female)	51,127 (52.5)
**Ethnicity**	
White	90,360 (92.8)
BAME	3056 (3.1)
Other	3997 (4.1)
**Height,** Mean ± SD	1.6 ± 0.1
**Weight,** Mean ± SD	74.4 ± 20.8
**Medication**	
Apixaban	82,073 (84.3)
Dabigatran	1125 (1.1)
Edoxaban	366 (0.3)
Rivaroxaban	13,849 (14.2)
**Treatment days**, Mean ± SD	513.9 ± 462.0
**Treatment years**	
≤ 1 year	48,054 (49.3)
2 years	18,894 (19.4)
3 years	16,316 (16.7)
4 years	10,058 (10.3)
> 4 years	4091 (4.2)
**BMI**, Mean ± SD	27.2 ± 7.2
**BMI category**	
Underweight	5525 (5.7)
Normal weight	37,316 (38.3)
Overweight	27,140 (27.9)
Obese	23,157 (23.8)
Morbidly obese	4275 (4.4)
**Comorbidity**	82,032 (84.2)
**Bleeding risk**	69,886 (71.7)
**VTE risk**	96,916 (99.5)

**Fig. 1:**
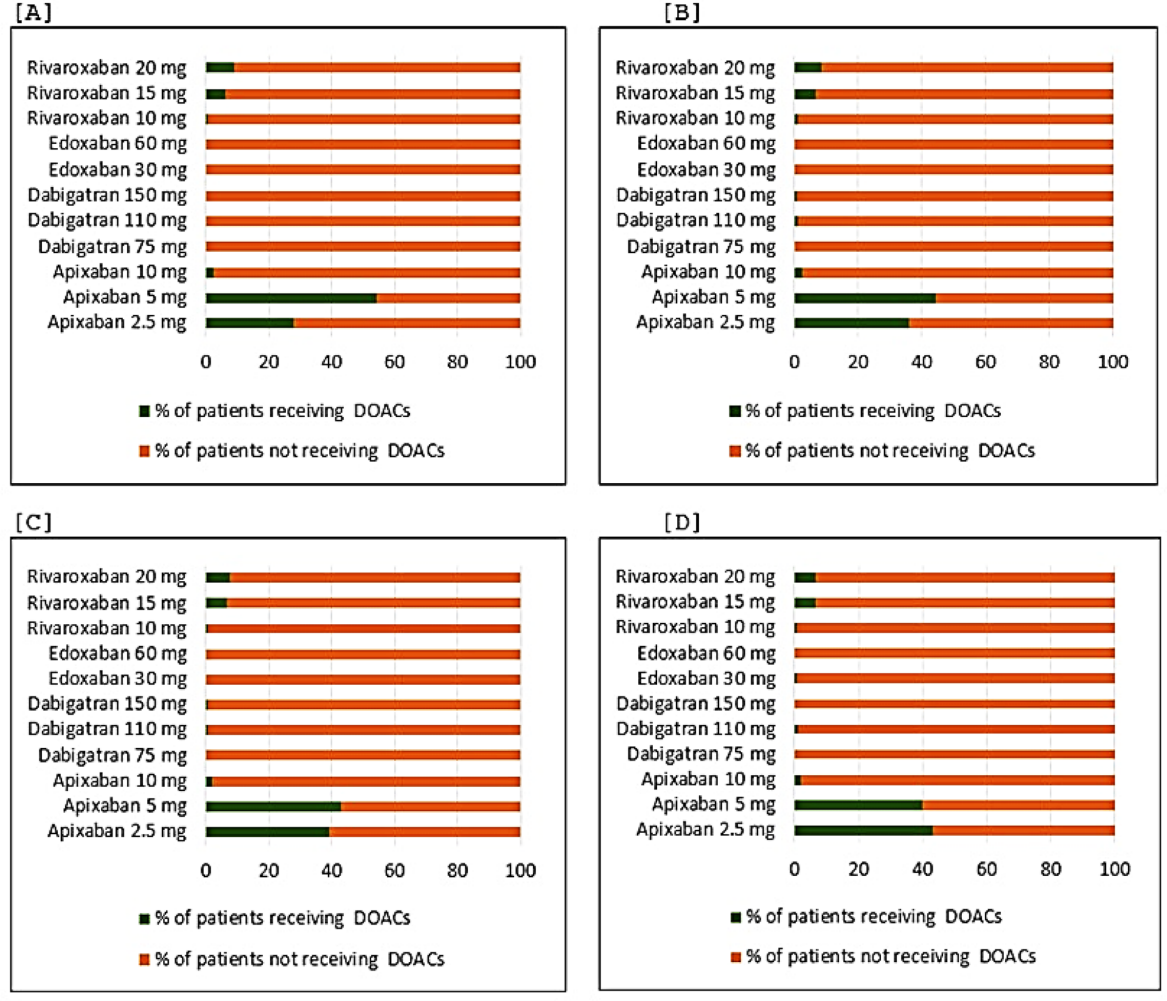
Daily doses of DOACs prescribed by BMI categories, **A** Morbidly obese patients, **B** Obese patients, **C** Overweight patients, **D** Normal weight patients

Apixaban and rivaroxaban were the two most prescribed DOACs in our study. Notably, prescribing of apixaban 5 mg daily in morbidly obese patients was the highest (54.3%) among the DOACs compared with patients in other BMI groups (Fig. 1).

The most essential and decisive features of the decision tree are DOACs treatment days, patient’s age, gender, and obesity status. Of these, treatment days, age, gender, and bleeding risk were the key drivers for predicting patient mortality. The input feature’s importance and contribution analysis increased the trust in the decision-making of the selected classifiers. Both models learned correct patterns from the dataset and correctly predicted the mortality (Fig. S5). Please refer to the Supplementary information for further information on key determinators.

As shown in Figs. S1–S4 (see Supplementary information), the decision trees and random forest ML algorithms, respectively, ranked other features (predictors)—aside from DOAC type—according to how strongly they impacted the patient’s clinical outcomes. From highest to lowest impact, the top four features impacting all-cause mortality based on both ML algorithms were: treatment days, length of stay, patient age, and the number of past emergency visits. These remained the same for other outcomes besides mortality (Supplementary figures).

## Body weight and clinical outcomes

High all-cause mortality rates were observed in the first year of DOAC treatment for normal weight (74%), obese (63%), and morbidly obese BMI classes (57%), respectively (Table S4). The mean count of emergency visits peaked in the second year of DOAC treatment for the normal weight, obese, and morbidly obese BMI categories.

A total of 811 CRNMB events were reported (811/97,413), with 246 events (30.3%) in overweight, 38 events (4.7%) in obese patients, and 527 events (65.0%) in normal-weight patients. Furthermore, 70 deaths were recorded due to CRNMB in the overweight cohort (roughly 93% of these deaths were associated with 5 mg apixaban), no deaths were recorded in the obese cohort, and 447 deaths occurred among the normal BMI patients (84% of the deaths due to 5 mg apixaban). Interestingly, no CRNMB events were reported in morbidly obese and underweight patients. In addition, the majority of these CRNMB events occurred within the first 2 years of DOACs treatment (515/811), with 0 events occurring after the fifth year of treatment.

The multivariate logistic regression results expressed in odds ratio (OR) are outlined in Table 2. Except for any thromboembolic events, the odds of adverse consequences, such as CRNMB events, ischaemic stroke, all-cause mortality, and prolonged hospital stay, were lower in patients in higher weight categories than normal weight patients, irrespective of DOAC type. For example, the odds of ischaemic stroke (OR 0.42, 95% CI: 0.36–0.88, *p* = 0.001) and all-cause mortality (OR 0.87, 95% CI: 0.81–0.95, *p* = 0.001) were lower in morbidly obese patients compared to normal body weight patients. The only exception is that thromboembolic event odds were higher in morbidly obese patients than in normal-weight patients (OR 3.63, 95% CI: 2.82–4.68, *p* = 0.001). There were no CRNMB events reported in morbidly obese patients. Except for ischaemic stroke, the odds of thromboembolic events, all-cause mortality, prolonged hospital stay, and emergency hospital visits were higher in underweight patients than in normal-weight patients.

**Table 2:** Results of multivariate logistic regression analysis (showing the likelihood of the outcomes (odds ratio) for the BMI categories

**BMI Category**	**OR**	**95% CI**		***p*-value**
		**Lower**	**Upper**	
**All-cause mortality**				
Morbidly obese (≥ 40)	0.78	0.72	0.84	0.001
Obese (30–39.9)	0.78	0.74	0.81	0.001
Overweight (24.5–29.9)	0.82	0.79	0.85	0.001
Underweight (0.0–18.49)	1.66	1.56	1.77	0.001
**Ischaemic stroke**				
Morbidly obese (≥ 40)	0.23	0.19	0.27	0.001
Obese (30–39.9)	0.82	0.78	0.86	0.001
Overweight (24.5–29.9)	0.88	0.84	0.92	0.001
Underweight (0.0–18.49)	0.53	0.49	0.58	0.001
**Any thromboembolic events**				
Morbidly obese (≥ 40)	4.03	3.12	5.20	0.001
Obese (30–39.9)	0.84	0.69	1.02	0.076
Overweight (24.5–29.9)	1.08	0.92	1.27	0.346
Underweight (0.0–18.49)	1.87	1.41	2.49	0.001
**Length of stay**				
Morbidly obese (≥ 40)	0.58	0.54	0.64	0.001
Obese (30–39.9)	0.51	0.49	0.54	0.001
Overweight (24.5–29.9)	0.65	0.62	0.68	0.001
Underweight (0.0–18.49)	1.52	1.37	1.70	0.001
**At least one emergency visit**				
Morbidly obese (≥ 40)	1.41	1.30	1.54	0.001
Obese (30–39.9)	1.13	1.08	1.17	0.001
Overweight (24.5–29.9)	1.02	0.98	1.06	0.266
Underweight (0.0–18.49)	1.19	1.11	1.29	0.001
**CRNMB**				
Normal weight (18.5–24.9)	-	-	-	-
Morbidly obese (≥ 40)	-	-	-	-
Obese (30–39.9)	0.11	0.08	0.15	0.001
Overweight (24.5–29.9)	0.70	0.60	0.82	0.001
Underweight (0.0–18.49)	-	-	-	-

Referent = Normal body weight (18.5–24.9)

*P*-value cut-off was 0.001

Variables adjusted for bleeding: ethnic group, age, gender, comorbidity, CKD, DOAC Type, thromboembolic events, length of stay, stroke, mortality, treatment years, and emergency admissions

Variables adjusted for stroke: ethnicity, age, length of stay, comorbidity, CKD, DOAC Type, emergency visits, thromboembolic events, treatment years, mortality, and bleeding

Variables adjusted for thromboembolic event: age, ethnicity, gender, comorbidity, DOAC Type, CKD, length of stay, emergency admissions, treatment years, mortality, bleeding, and stroke

Variables adjusted for mortality: age, ethnicity, gender, comorbidity, CKD, DOAC Type, length of stay, emergency admissions, treatment years, thromboembolic events, bleeding risk, and stroke

Variables adjusted for emergency visits: ethnicity, age, length of stay, comorbidity, CKD, DOAC Type, thromboembolic events, treatment years, stroke mortality, and bleeding

Variables adjusted for LoS: age, ethnicity, comorbidity, CKD, DOAC Type, length of stay, emergency admissions, treatment years, thromboembolic events, bleeding, and stroke

## DOAC type and clinical outcomes

The association between DOAC type and clinical outcomes (Tables S4 and 3) was derived based on multivariate logistic regression analysis and Kaplan–Meier analysis. Apixaban significantly lowered the odds of all-cause mortality (OR 0.88, 95% CI: 0.84–0.91, *p* = 0.001), prolonged hospital stays (OR 0.89, 95% CI: 0.85–0.94, *p* = 0.001), emergency visits (OR 0.68, 95% CI: 0.65–0.72, *p* = 0.001) and any thromboembolic events (OR 0.38, 95% CI: 0.33–0.44, *p* = 0.001), respectively. On the other hand, the odds of CRNMB (OR 1.48, 95% CI: 1.18–1.84, *p* = 0.001) and ischaemic stroke events (OR 2.07, 95% CI: 1.95–2.19, *p* = 0.001) were higher with apixaban. There was no consistent trend in bleeding cases observed across the 5 years of treatment, but ischemic stroke cases rose for the first 4 years of treatment.

**Table 3:** Association between specific DOAC therapy and clinical outcomes irrespective of body weight

**Outcome**	**DOAC**	**OR**	**95% Confidence Interval**		
			**Lower**	**Upper**	***p*-value**
**Ischaemic stroke**	Apixaban	2.07	1.95	2.19	0.001
	Rivaroxaban	0.47	0.45	0.50	0.001
	Edoxaban	0.95	0.71	1.27	0.742
	Dabigatran	0.17	0.13	0.24	0.001
**All-cause mortality**	Apixaban	0.88	0.84	0.91	0.001
	Rivaroxaban	1.10	1.06	1.15	0.001
	Edoxaban	0.43	0.34	0.53	0.001
	Dabigatran	2.26	1.95	2.62	0.001
**Any thromboembolic event**	Apixaban	0.38	0.33	0.44	0.001
	Rivaroxaban	2.82	2.42	3.27	0.001
	Edoxaban	2.08	0.96	4.50	0.062
**CRNMB**	Apixaban	1.48	1.18	1.84	0.001
	Rivaroxaban	0.76	0.61	0.95	0.014
**At least one emergency visit**	Apixaban	0.68	0.65	0.72	0.001
	Rivaroxaban	1.45	1.38	1.52	0.001
	Edoxaban	1.05	0.81	1.35	0.713
	Dabigatran	1.63	1.36	1.95	0.001
**Length of stay** **(more than a week)**	Apixaban	0.89	0.85	0.94	0.001
	Rivaroxaban	1.22	1.16	1.29	0.001
	Edoxaban	0.97	0.70	1.34	0.837
	Dabigatran	0.69	0.59	0.81	0.001

Emergency visit was no visit (referent) and one or more one visits. Length of stay was less than week stay (referent), and more than week stay

Covariates were selected based on their contribution to a particular outcome (based on ML technique) as indicated in Figs. S1–S5

With rivaroxaban, there were significantly higher odds of all-cause mortality (OR 1.10, 95% CI: 1.06–1.15, *p* = 0.001), prolonged length of hospital stays (OR 1.12, 95% CI: 1.16–1.29, *p* = 0.001) (agreeing with the rise in cases for stays of more than 1 week across the first 4 years of treatment), one or more emergency visits (OR 1.45, 95% CI: 1.38–1.52, *p* = 0.001) and thromboembolic events (OR 2.82, 95% CI: 2.42–3.27, *p* = 0.001). However, there were lower odds of ischemic stroke (OR 0.47, 95% CI: 0.45–0.50, *p* = 0.001) following treatment with rivaroxaban though it did not reflect the increase in the proportion of cases across the treatment years.

The only significant impact edoxaban has was on the odds of all-cause mortality (OR 0.43, 95% CI: 0.34–0.53, *p* = 0.001), which decreased. This agrees with the declining proportion of mortalities across the 3 years of treatment. Dabigatran significantly lowered the odds of ischemic strokes (OR 0.17, 95% CI: 0.13–0.24, *p* = 0.001) and prolonged length of stay (OR 0.69, 95% CI: 0.59–0.81, *p* = 0.001) but raised the odds of all-cause mortality (OR 2.26, 95% CI: 1.95–2.62, *p* = 0.001) and one or more emergency visits (OR 1.63, 95% CI: 1.36–1.95, *p* = 0.001).

**Fig. 2:**
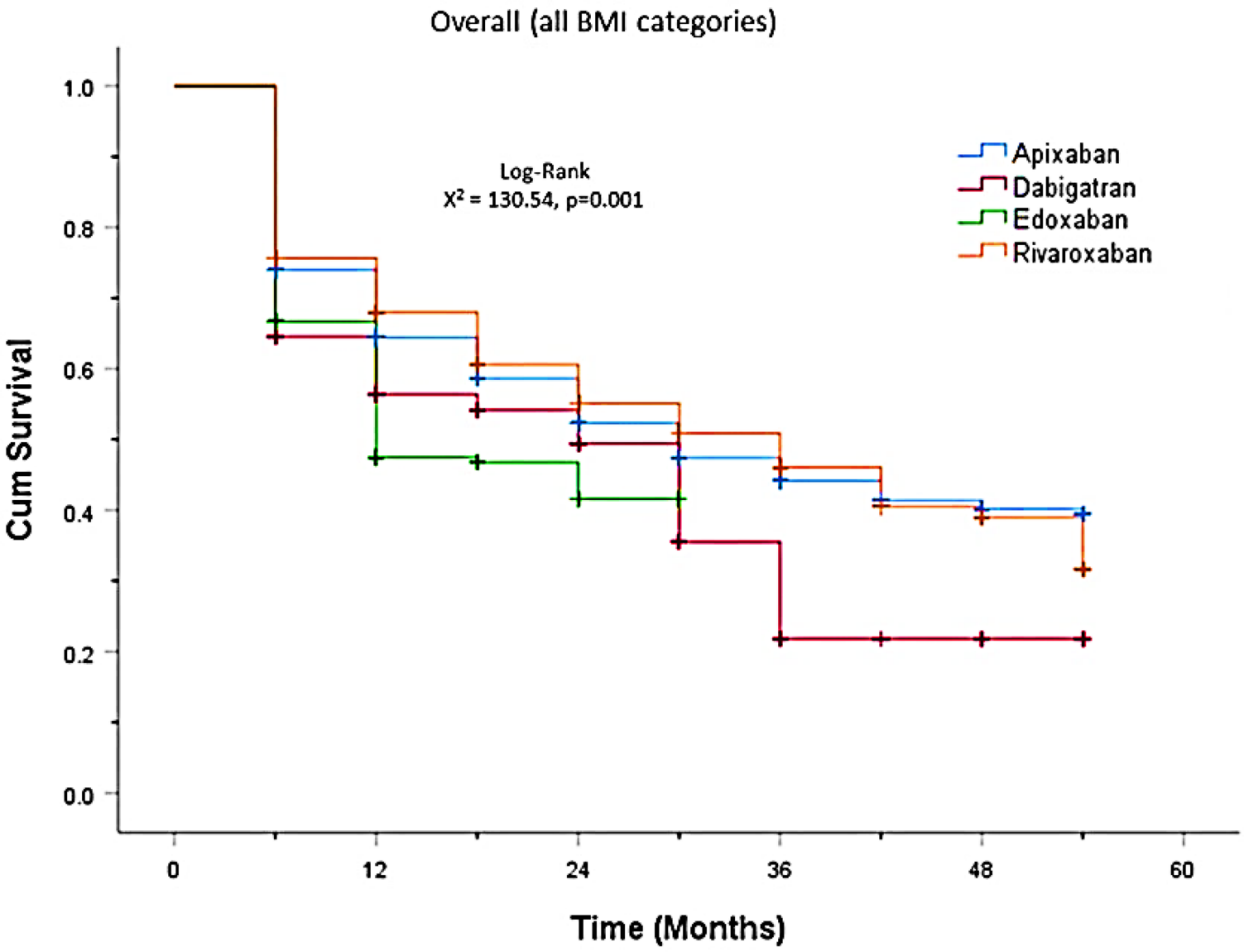
Plot of cumulative mortality rates stratified by DOACs administration

**Fig. 3:**
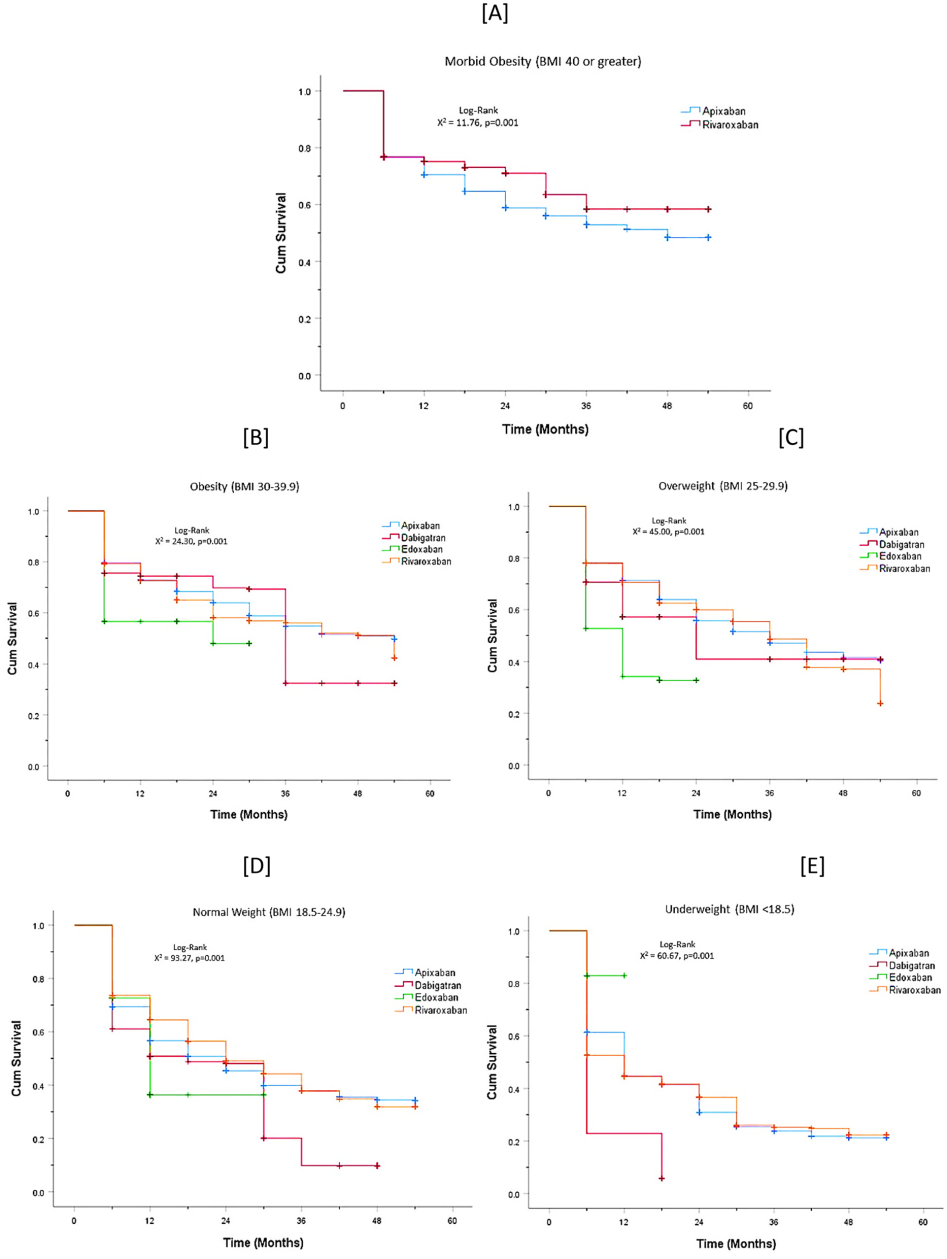
Plot of cumulative mortality rates stratified by DOACs administration; all lines have the same meaning as the labels in Fig. 1A. **A** Morbid obesity; **B** obesity; **C** overweight; **D** normal weight; **E** underweight

Kaplan–Meier survival analysis showed a higher rate of mortality in the dabigatran and rivaroxaban groups than in the apixaban and edoxaban groups (log-rank *p* < 0.001, Fig. 2), regardless of the obesity status. In the morbid obesity subgroup (A), the rate of mortality was higher in the apixaban group than in the rivaroxaban group (log-rank *p* = 0.001, Fig. 3). However, in the obesity (B) and overweight (C) subgroups, the rate of mortality was lower in the apixaban group than in the rivaroxaban group (log-rank *p* = 0.001, *p* = 0.001, Fig. 3).

## Discussion

This large, single-centre study explores a unique perspective on the safety and efficacy aspects of individual DOACs by analysing a large real-world dataset for patients in different body weight classes. Overall, the results indicate a positive impact of specific DOAC types on specific clinical outcomes, with significant differences between obese and morbidly obese subgroups and other BMI categories.

The association between the BMI category (of the patients on DOACs) and the clinical outcomes was mixed. For example, being underweight significantly raised the odds of any thromboembolic events, all-cause mortality, length of stay, and emergency visits—the odds of ischemic stroke dropped significantly in the underweight patients (OR 0.53, 95% CI: 0.49–0.58, *p* = 0.001). The negative impact on a safety outcome (all-cause mortality) was expected as it reflects the higher exposure of standard dose DOACs that increase the risk of adverse events. Other factors besides high exposure, like age and comorbidity, may be responsible for the poor efficacy outcomes. A morbidly obese BMI was associated with significantly lower odds of ischemic stroke, length of stay, and all-cause mortality but higher odds of thromboembolic events. The positive outcomes may be attributed to other factors, including obesity paradox and DOAC type or dose—obesity paradox describes a phenomenon in which clinical outcomes like mortality appear to improve with high BMI (e.g., morbid obesity). In contrast, the negative outcome (e.g., thromboembolic events) may reflect underexposure given the pharmacokinetic or pharmacodynamic changes because of obesity (pharmacokinetic changes include increased clearance due to higher metabolic enzyme activity and glomerular filtration rate). Interestingly, the obese BMI category was associated with significantly lower odds of bleeding as expected.

The results from this study agreed with some of the previously published evidence: for example, Whittemore et al. [7], analysed the adjusted odds ratio following multivariate logistic regression and revealed that increased body weight decreased the risk of bleeding events. Wu et al. [24], also found that a higher BMI was negatively associated with bleeding events and mortality compared to normal BMI for DOACs overall (i.e., dabigatran and rivaroxaban). This suggests that DOACs were linked to better survival and lower bleeding risk in the obese (higher BMI) cohort compared to normal BMI patients. This was not the case for thromboembolic events, as no statistically significant association was reported for the morbidly obese cohort. Also, subgroup analysis of rivaroxaban and dabigatran did not yield statistically significant associations.

Furthermore, using a Cox proportional hazards model, Weitz et al. [25], reported a lower risk of all-cause mortality in obesity compared to patients with normal BMI. This suggests better safety and effectiveness (obesity paradox). On the other hand, Briasoulis et al. [26], reported an increased risk of all-cause mortality (HR of 1.12, 95% CI: 1.02–1.23) but no significant difference in stroke and bleeding events for morbidly obese patients when apixaban was compared with rivaroxaban. Also, in the subgroup of individuals with morbid obesity, dabigatran was associated with significantly lower all-cause mortality compared to rivaroxaban; apixaban was associated with greater mortality than dabigatran and rivaroxaban.

Meanwhile, in the studies by Netely et al. [11], and Wang et al. [19], the obese BMI category had no significant impact on bleeding or thrombotic events, agreeing with the findings from Aloi et al. [16], Perino et al. [17], and Deitelzweig et al. [18]. The authors suggested their findings were due to confounding biases associated with most observational studies. Specifically, other observational studies and randomised controlled trials (RCTs) widely suggest that increased BMI in VTE/NVAF has no significant effect on the safety and efficacy of apixaban. In other words, despite exposure being slightly reduced, no dose adjustment was required [20]. However, this does not dismiss the claim that the morbidly obese category had the most incidence of thrombotic events among the BMI classes [8, 9, 21, 22].

Lucijanic et al. [13], established that obesity increased the risk (odds) of stroke and bleeding in DOACs overall: dabigatran conferring lower efficacy (higher thrombosis/stroke risk) and Factor Xa inhibitors (rivaroxaban, edoxaban, and apixaban) increasing the odds of bleeding (somewhat similar to our result). Their study observed a positive association between BMI and time to thrombosis (TTT), and time to bleeding (TTB) for DOACs. Potential factors responsible for the increased risk of bleeding were inappropriate dosage regimens and concomitant interacting medications [23]. It is important to note that reference to specific DOAC doses was not made in the study (absence of subgroup analysis).

In our study, the majority of the patients were on apixaban (84%, *n* = 82,073), similar to the study by Briasoulis et al. [26]. This reinforces the status of apixaban as the most prescribed DOAC in the NHS (at the time of this study). In addition, the mean age in our study is within the same range as the study carried out by Barakat et al. [1], (74.1 years), which also examined the outcomes of DOACs in patients across different BMI categories—DOACs were linked to a lower risk of bleeding and stroke among obese and morbidly obese patients.

Notably, our study was based on the assumption that bleeding and mortality were measures for safety during ischaemic stroke or thromboembolic events, emergency visits and length of stay were used as measures for effectiveness—for instance, the shorter length of stay (or fewer emergency admissions) implies positive outcomes. A possible explanation for the inverse relationship with BMI for edoxaban could be due to the link with positive outcomes (which may include low risk of bleeding) from treatment and hence early discharge—or the sample size of patients (on edoxaban) was insufficient to draw firm conclusions. It is worth mentioning that bleeding events occurred only in normal BMI and overweight and obese patients, respectively. No deaths were recorded for obese patients who experienced bleeding.

Our results add weight to the potential safety and effectiveness of specific DOAC types (for example, edoxaban) in different patient BMI groups, even though the positive clinical outcomes were inconsistent across DOAC types or BMI groups. Indeed, there is mounting evidence of the safety and effectiveness of DOACs in obese patients, which is comparable to or even better compared to normal-weight patients [3].

A key strength of this study was the inclusion of a large sample size of obese patients who were underrepresented in landmark DOAC trials and remained underappreciated in clinical practice. Machine learning (ML) models are data-driven (i.e., thrive on large datasets) and known for their excellent pattern recognition and predictive abilities. The performance of the ML model relies heavily on the quality of data. The importance of input features towards class attribute prediction in decision trees and random forests increased the confidence in our ML solution. The ML approach is a multifaceted technique where the outcome goals could vary from task to task. Apart from predicting the unknown data, a well-trained and accurate ML model could be used to analyse the critical features in the dataset.

Irrespective of the ML model’s strength, low-quality or noisy data will result in poor predictions. Furthermore, a limitation of the study was its retrospective design hence the susceptibility to confounding biases. Also, there was sampling bias: it was impractical to do regression analysis on dabigatran and edoxaban (by BMI category) due to insufficient sample size (as a result of limited prescribing); the samples for bleeding outcomes were highly imbalanced (with the majority of cases being positive). It is also important to acknowledge that by grouping patients together as per outcomes, our analyses did not quantitatively account for their differential risks of adverse clinical outcomes (e.g., CHA2DS2-VASc score for stroke and HAS-BLED score for bleeding risk); also, there was no justification for recommendation of DOAC based on BMI thresholds. Finally, our use of BMI as a body size descriptor may limit the applicability of our results in terms of dose determination (it fails to account for the distribution of body fat given that DOACs are lipophilic)—more accurate biomarkers that describe the pharmacokinetics of DOACs are required (perhaps waist-to-hip ratio).Undoubtedly, these limitations may undermine the strength of our findings and reemphasise the importance of more accurate (advanced) models and well-designed studies (e.g. randomised controlled trials).

## Conclusion

This large, single-centre study shows the positive effects of DOAC therapy on clinical outcomes, particularly in patients with high body weight. The key determinants for study outcomes were treatment days, age, and bleeding risk—obesity and DOAC type ranked relatively low. The findings from our study—including conclusions from reviewed literature—imply the use of DOACs in obese and morbidly obese patients does not pose a significant safety threat and has demonstrated satisfactory effectiveness. This gives clinicians good assurance when prescribing medications like apixaban and rivaroxaban. This implies that more morbidly obese patients in NHS would benefit from DOACs without the constraints of monitoring assays that are in limited supply. However, this still needs validation by further studies such as prospective randomised controlled trials.

### Supplementary Information

Below is the link to the electronic supplementary material.

Supplementary file1 (DOCX 476 KB)

Supplementary file2 (DOCX 33 KB)

## Data Availability

The data that support the findings of this study are available on request from the corresponding author. The data are not publicly available due to privacy or ethical restrictions.
