# Supplementary material for: Direct oral anticoagulants and the risk of adverse clinical outcomes among patients with different body weight categories: a large hospital-based study
Source: Eur J Clin Pharmacol. 2023 Nov 18;80(1):163–73. doi: 10.1007/s00228-023-03593-2 (PMC10781787; doi:10.1007/s00228-023-03593-2)
Supplement: Supplementary file 1 — Supplementary file1 (DOCX 476 KB) [file 228_2023_3593_MOESM1_ESM.docx]

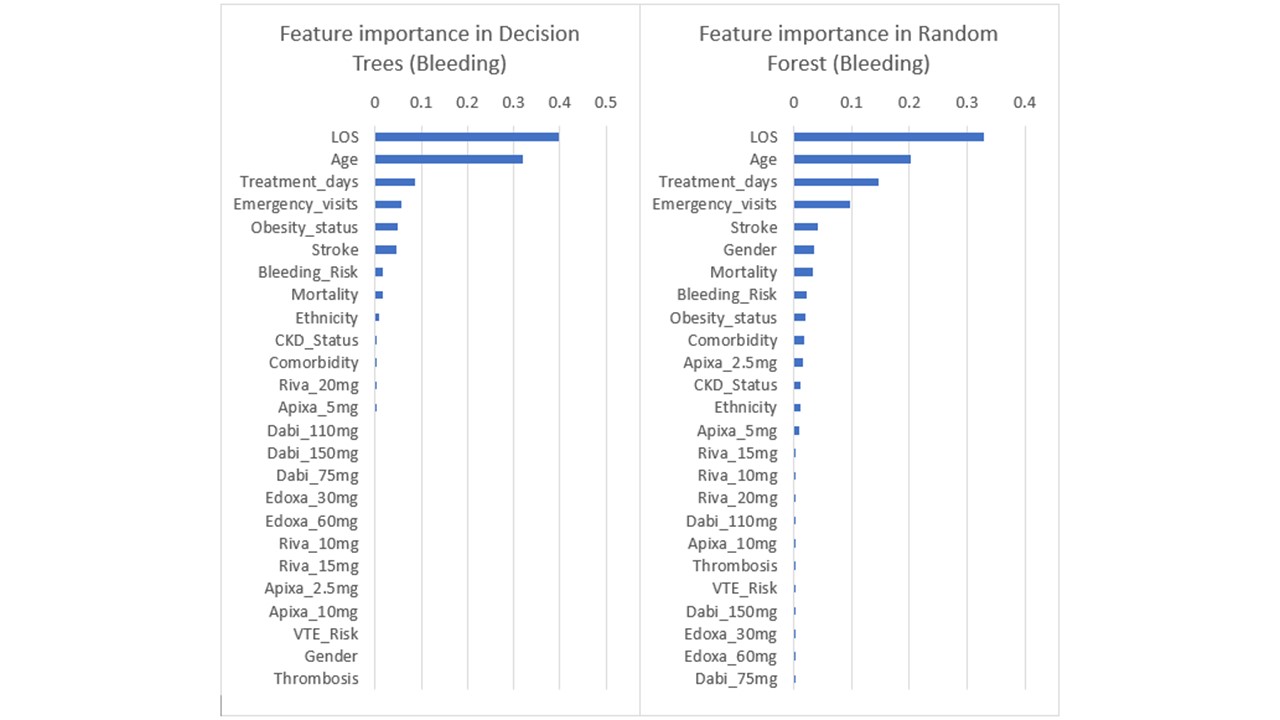


**Figure S1:** Ranking of features with the overall patient dataset according to their importance in contributing to bleeding using RF = Random Forest and DT = Decision Trees.


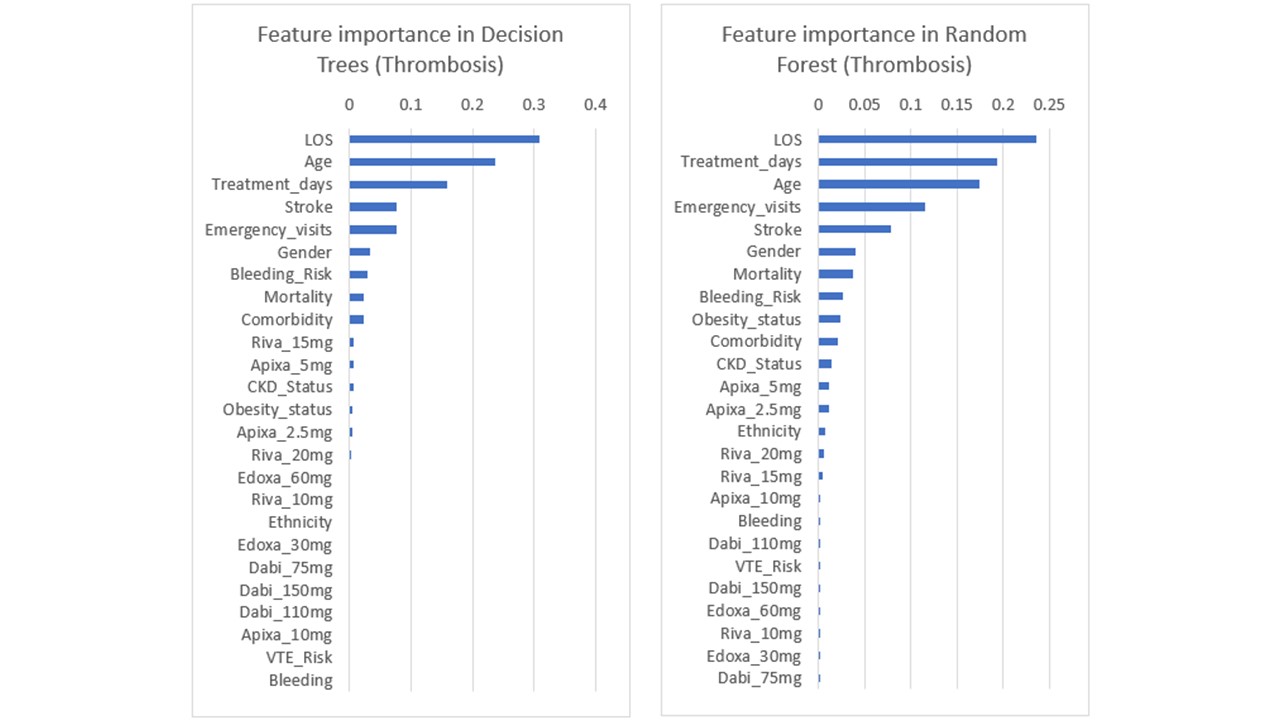


**Figure S2:** Ranking of features with the overall patient dataset according to their importance in contributing to thrombosis using RF = Random Forest and DT = Decision Trees.


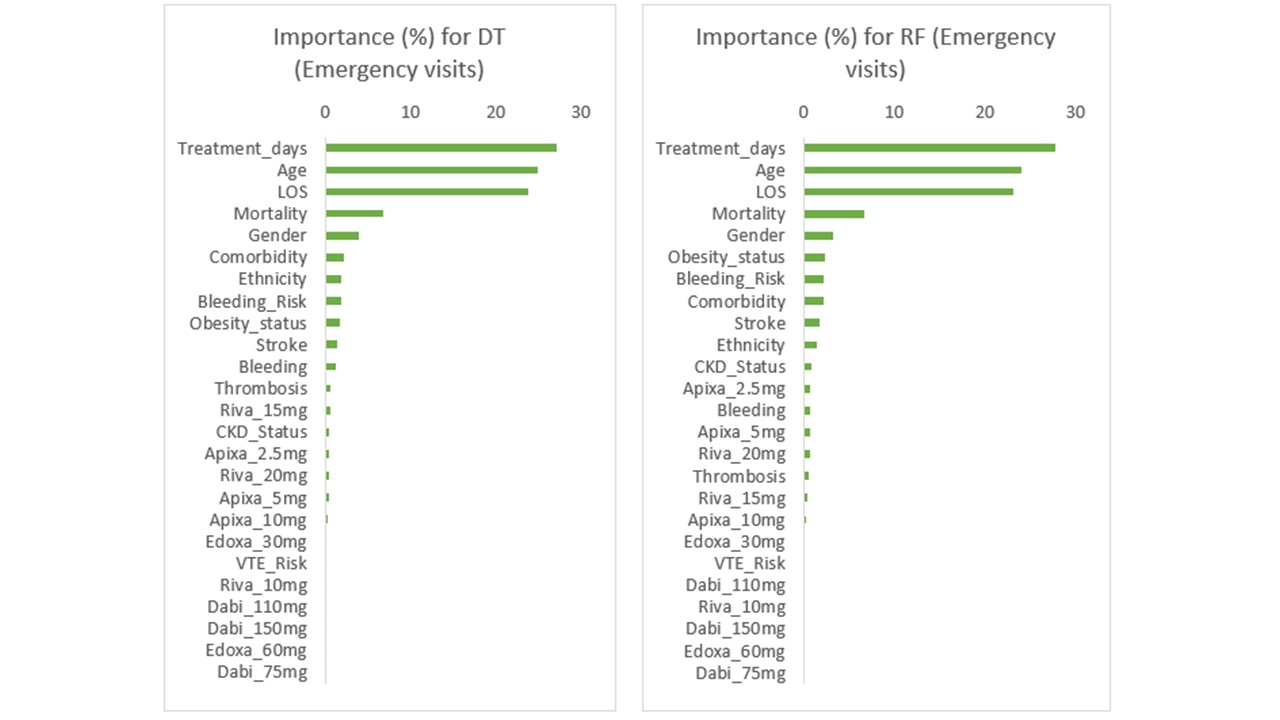


**Figure S3:** Ranking of features with the overall patient dataset according to their importance in contributing to emergency visits using RF = Random Forest and DT = Decision Trees.


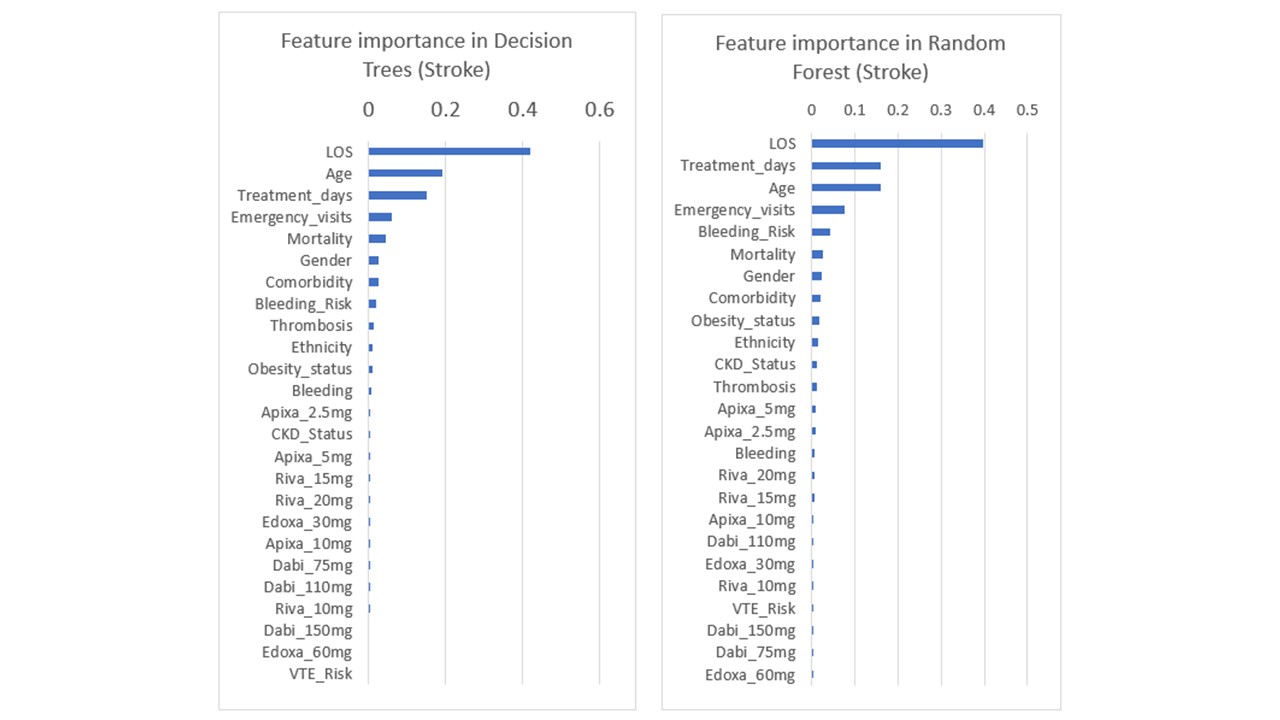


**Figure S4:** Ranking of features with the overall patient dataset according to their importance in contributing to stroke using RF = Random Forest and DT = Decision Trees.


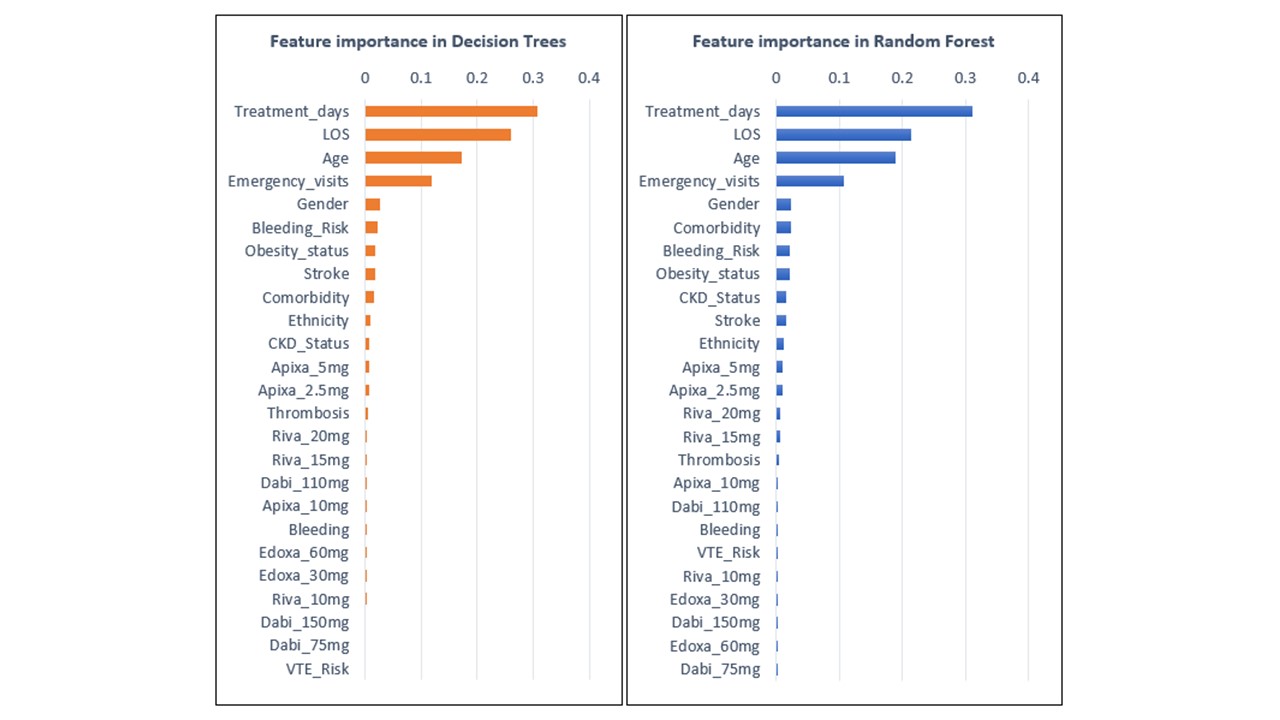


**Figure S5:** Ranking of features according to their importance in contributing to mortality using Random Forest and Decision Trees [LOS=length of strength; CKD=chronic kidney disease]
