# Supplementary material for: Direct oral anticoagulants and the risk of adverse clinical outcomes among patients with different body weight categories: a large hospital-based study
Source: Eur J Clin Pharmacol. 2023 Nov 18;80(1):163–73. doi: 10.1007/s00228-023-03593-2 (PMC10781787; doi:10.1007/s00228-023-03593-2)
Supplement: Supplementary file 2 — Supplementary file2 (DOCX 33 KB) [file 228_2023_3593_MOESM2_ESM.docx]

**Table S1:** Overall accuracy of each ML classifiers

| Machine learning algorithm | Accuracy |
| --- | --- |
| Decision Trees | **0.988** |
| k Nearest Neighbour | 0.972 |
| Random Forest | **0.992** |
| Logistic regression | 0.755 |
| Gradient Boosting classifier | 0.810 |
| Support vector machine | 0.810 |

**Table S2:** Performance metrics of decision trees and random forests on various side effects prediction

| **Performance Metrics →** | **Precision** | | **Recall** | | **F1-Score** | | **Support** | |
| --- | --- | --- | --- | --- | --- | --- | --- | --- |
| **Attributes ↓** | **DT** | **RF** | **DT** | **RF** | **DT** | **RF** | **DT** | **RF** |
| Mortality = 0 | 0.99 | 0.99 | 0.99 | 0.99 | 0.99 | 0.99 | 14,867 | 14,867 |
| Mortality = 1 | 0.99 | 0.99 | 0.99 | 0.99 | 0.99 | 0.99 | 14,357 | 14,357 |
| Bleeding = 0 | 0.97 | 1.00 | 1.00 | 1.00 | 0.99 | 1.00 | 243 | 243 |
| Bleeding = 1 | 1.00 | 1.00 | 1.00 | 1.00 | 1.00 | 1.00 | 28,981 | 28,981 |
| Stroke = 0 | 1.00 | 1.00 | 1.00 | 1.00 | 1.00 | 1.00 | 24,044 | 24,044 |
| Stroke =1 | 0.99 | 0.99 | 0.99 | 0.99 | 0.99 | 0.99 | 5180 | 5180 |
| Thrombosis = 0 | 1.00 | 1.00 | 1.00 | 1.00 | 1.00 | 1.00 | 28,934 | 28,934 |
| Thrombosis = 1 | 0.99 | 1.00 | 0.97 | 0.98 | 0.98 | 0.99 | 290 | 290 |

Note: DT: decision tree, RF, random forest

**Table S3:** Summary of outcomes according to normal weight and obesity

| BMI Cat | Treatment Years | LoS in days | Emergency visits | CRNMB Event | | Ischaemic stroke | | Any TE Event | | All-cause mortality | |
| --- | --- | --- | --- | --- | --- | --- | --- | --- | --- | --- | --- |
|  |  | **Mean (SD)** | **Mean (SD)** | **Yes**  **n (%)** | **No**  **n (%)** | **Yes**  **n (%)** | **No**  **n (%)** | **Yes**  **n (%)** | **No**  **n (%)** | **Yes**  **n (%)** | **No**  **n (%)** |
| Normal weight (18.5-24.9) | ≤1yr | 35.25 (29.13) | 1.65  (2.03) | 312  (1.5) | 20,258  (98.5) | 3,579  (17.4) | 16,991  (82.6) | 106  (0.5) | 20,464  (99.5) | 15,232  (74.0) | 5,338  (26.0) |
|  | 2yrs | 31.03  (27.18) | 3.08  (3.14) | 107  (1.6) | 6,710  (98.4) | 1,043  (15.3) | 5,774  (84.7) | 170  (2.5) | 6,647  (97.5) | 3,181  (46.7) | 3,636  (53.3) |
|  | 3yrs | 54.15  (60.08) | 3.07  (3.68) | 80  (1.4) | 5,579  98.6) | 1,827  (32.3) | 3,832  (67.7) | 40  (0.7) | 5,619  (99.3) | 1,753  (31.0) | 3,906  (69.0) |
|  | 4yrs | 41.02  (33.00) | 2.60  (3.27) | 28  (0.8) | 3,290  (99.2) | 1,209  (36.4) | 2,109  (63.6) | 41  (1.2) | 3,277  (98.8) | 376  (11.3) | 2,942  (88.7) |
|  | 5yrs | 47.05  (51.60) | 2.03  (2.43) | 0  (0.0) | 952  (100) | 345  (36.2) | 607  (63.8) | 2  (0.2) | 950  (99.8) | 4  (0.4) | 948  (99.6) |
| Obese  (30-39.9) | ≤1yr | 28.92  (21.91) | 1.75  (2.02) | 0  (0.0) | 9,680  (100) | 1293  (13.4) | 8,387  (86.6) | 61  (0.6) | 9,619  (99.4) | 6,134  (63.4) | 3,546  (36.6) |
|  | 2yrs | 26.42  (24.78) | 3.38  (3.85) | 23(0.5) | 4,575  (99.5) | 617  (13.4) | 3,981  (86.6) | 1  (0.0) | 4,597  (100) | 1,727  (37.6) | 2,871  (62.4) |
|  | 3yrs | 33.08  (34.06) | 2.99  (2.98) | 3  (0.1) | 4,266  (99.9) | 869  (20.4) | 3,400  (79.6) | 56  (1.3) | 4,213  (98.7) | 1,076  (25.2) | 3,193  (74.8) |
|  | 4yrs | 28.10  (34.78) | 2.61  (3.36) | 12  (0.4) | 3,037  (99.6) | 435  (14.3) | 2,614  (85.7) | 56  (1.8) | 2,993  (98.2) | 320  (10.5) | 2,729  (89.5) |
|  | 5yrs | 18.32  (22.14) | 1.63  (2.47) | 0  (0.0) | 1,561  (100) | 264  (16.9) | 1,297  (83.1) | 0  (0.0) | 1,561  (100) | 59  (3.8) | 1,502  (96.2) |
| Morbidly Obese  (≥40) | ≤1yr | 23.63  (17.44) | 1.85  (1.69) | 0  (0.0) | 2,081  (100) | 118  (5.7) | 1,963  (94.3) | 17  (0.8) | 2,064  (99.2) | 1,185  (56.9) | 896  (43.1) |
|  | 2yrs | 20.31  (16.12) | 3.99  (3.85) | 0  (0.0) | 886  (100) | 25  (2.8) | 861  (97.2) | 6  (0.7) | 880  (99.3) | 296  (33.4) | 590  (66.6) |
|  | 3yrs | 17.94  (15.83) | 2.65  (2.94) | 0  (0.0) | 615  (100) | 69  (11.2) | 546  (88.8) | 69  (11.2) | 546  (88.8) | 134  (21.8) | 481  (78.2) |
|  | 4yrs | 14.69  (18.67) | 2.51  (3.74) | 0  (0.0) | 532  (100) | 47  (8.8) | 485  (91.2) | 0  (0.0) | 532  (100) | 33  (6.2) | 499  (93.8) |
|  | 5yrs | 7.37  (6.36) | 2.48  (3.13) | 0  (0.0) | 161  (100) | 0  (0.0) | 161  (100) | 0  (0.0) | 161  100) | 0  (0.0) | 161  (100) |

**Table S4:** Summary of outcomes according to specific DOAC type

| DOACs | Treatment Years | LoS | | Emergency visits | | CRNMB | | Ischaemic Stroke | | Any TE event | | All-cause mortality | |
| --- | --- | --- | --- | --- | --- | --- | --- | --- | --- | --- | --- | --- | --- |
|  |  | **> 1 week**  **n (%)** | **≤ 1 week**  **n (%)** | **Yes**  **n (%)** | **No**  **n (%)** | **Yes**  **n (%)** | **No**  **n (%)** | **Yes**  **n (%)** | **No**  **n (%)** | **Yes**  **n (%)** | **No**  **n (%)** | **Yes**  **n (%)** | **No**  **n (%)** |
| Apixaban | ≤1yr | 36,628 (89.1) | 4,474  (10.9) | 29,906 (72.8) | 11,196 (27.2) | 385  (0.9) | 40717 (99.1) | 6783  (16.7) | 34229 (83.3) | 364  (0.9) | 40738 (99.1) | 28233  (68.7) | 12869  (31.3) |
|  | 2yrs | 13,064 (83.4) | 2,604  (16.6) | 13,736 (87.7) | 1,932 (12.3) | 108  (0.7) | 15560 (99.3) | 2736  (17.5) | 12932 (82.5) | 112  (0.7) | 15556 (99.3) | 7221  (46.1) | 8447  (53.9) |
|  | 3yrs | 10,981  (80.5) | 2,652  (19.5) | 10,463 (76.7) | 3,170 (23.3) | 152  (1.1) | 13481 (98.9) | 3417  (25.1) | 10216 (74.9) | 157  (1.2) | 13476 (98.8) | 3619  (26.5) | 10014  (73.5) |
|  | 4yrs | 5,218 (63.3) | 3,030  (36.7) | 5,616 (68.1) | 2,632 (31.9) | 50  (0.6) | 8198  (99.4) | 2005  (18.0) | 6243 (75.7) | 44  (0.5) | 8204  (99.3) | 955  (11.6) | 7239  (88.4) |
|  | 5yrs | 1,676 (49.0) | 1,746  (51.0) | 2,029 (59.3) | 1,393 (40.7) | 0  (0.0) | 3422  (100) | - | - | 18  (0.5) | 3404  (99.1) | 59  (1.7) | 3363  (98.3) |
| Rivaroxaban | ≤1yr | 5,196 (85.6) | 872  (14.4) | 4,814 (79.3) | 1,254 (20.7) | 0  (0.0) | 6068  (100) | 357  (5.9) | 5711 (94.1) | 82  (1.4) | 5986  (98.6) | 4320  (71.2) | 1748  (28.8) |
|  | 2yrs | 2,560 (84.0) | 488  (16.0) | 2,830 (92.8) | 218  (7.2) | 22  (0.7) | 3026  (99.3) | 232  (7.6) | 2816 (92.4) | 88  (2.9) | 2960  (97.1) | 1408  (46.2) | 1640  (53.8) |
|  | 3yrs | 1,998 (82.5) | 423  (17.5) | 2,126 (87.8) | 295 (12.2) | 66  (2.7) | 2355  (97.3) | 478  (19.7) | 1943 (80.3) | 41  (1.7) | 2380  (98.3) | 660  (27.3) | 1761  (72.7) |
|  | 4yrs | 1,372 (81.6) | 310  (18.4) | 1,388 (82.5) | 294 (17.5) | 28  (1.7) | 1654  (98.3) | 216  (12.8) | 1466 (87.2) | 53  (3.2) | 1629  (96.8) | 332  (19.7) | 1350  (80.3) |
|  | 5yrs | 556 (88.3) | 74  (11.7) | 517 (82.1) | 113 (17.9) | 0  (0.0) | 630  (100) | 231  (36.7) | 399  (63.3) | 0  (0.0) | 630  (100) | 117  (18.6) | 513  (81.4) |
| Edoxaban | ≤1yr | 275 (92.6) | 22  (7.4) | 208 (70.0) | 89  (30.0) | 297  (100) | - | 60  (20.2) | 237  (79.8) | 0  (0.0) | 297  (100) | 160  (53.9) | 137  (46.1) |
|  | 2yrs | 27  (57.4) | 20  (42.6) | 46 (97.9) | 1  (2.1) | 47  (100) | - | 2  (4.3) | 45  (95.7) | 7  (14.9) | 40  (85.1) | 5  (10.6) | 42  (89.4) |
|  | 3yrs | 15  (68.2) | 7  (31.8) | 22  (100) | 0  (0.0) | 22  (100) | - | 0  (0.0) | 22  (100) | 0  (0.0) | 22  (100) | 0  (0.0) | 22  (100) |
| Dabigatran | ≤1yr | 477 (81.3) | 110  (18.7) | 486 (82.8) | 101 (17.2) | 587  (100) | - | 36  (6.1) | 551  (93.9) | - | 587  (100) | 486  (82.8) | 101  (17.2) |
|  | 2yrs | 89  (67.9) | 42  (32.1) | 131 (100) | 0  (0.0) | 131  (100) | - | 7  (5.3) | 124  (94.7) | - | 131  (100) | 60  (45.8) | 71  (54.2) |
